# Eye of the beholder—Tales from Prostate Cancer Active Surveillance Development

**DOI:** 10.1002/bco2.80

**Published:** 2021-03-27

**Authors:** John W. Davis

**Affiliations:** ^1^ Editor—BJUI Compass

For the March 2021 issue of *BJUI Compass*, we have our first paper on active surveillance for prostate cancer—a major part of my clinical practice. I have been around long enough to have observed this idea start as a research concept that needed IRB protocol and informed consent protections, to being part of guidelines‐based clinical practice. Meanwhile, the practice is an important part of how we manage prostate cancer screening efforts that yield low‐grade cancer. The tools for effective screening and active surveillance have grown and have significant overlap—secondary biomarkers, MRI imaging, fusion or transperineal biopsy, etc.

A favorite memory from my early practice years was from the Winter meeting of the Society of Urologic Oncology in 2005 (I think) when it was a smaller research‐oriented retreat. We had back‐to‐back speakers on prostate cancer with different messages that had a bit of humorously conflicting conclusions. The first speaker was Andrew Vickers from Memorial Sloan Kettering Cancer Center,^1^ presenting on the learning curve of radical prostatectomy. At the end of that lecture, everyone was probably thinking to themselves: “Oh no, I need to go home and double my radical prostatectomy case volumes, or I might be labelled as a low volume surgeon.” The next speaker was Lawrence Klotz from the University of Toronto^2^ who presented his maturing data and successes with active surveillance for low‐risk prostate cancer. At the end of that lecture, everyone was probably thinking to themselves: “Oh no, I need to go home and cut my radical prostatectomy volumes in half, or I might be labelled as an over‐treating/patient‐harming surgeon.” This era of active surveillance data was a breakthrough in the field—previous presentations of less mature data were often attacked for being dangerous and risking pT3 cancer progression. This lecture was one of the early ones that was mature enough to be convincing that low‐risk prostate cancer was a different entity.

Of course, by 2021, we now have strong guidance on the surveillance management of low‐risk prostate cancer and can focus treatments more on intermediate to high‐risk patients. In support of Dr Vickers findings, we have trended towards more surgical sub‐specialization and higher case volumes to improve quality of results. In this same area of case selection and referrals, however, there are low and favourable‐intermediate risk patients who are appropriate candidates for active surveillance, but can be predicted to be at high risk of progression and need for future treatment. These patients are an interesting group to talk with, as when you spell out the choices and bigger picture, you often get patients gravitating towards one of the two ways of thinking about it: (1) treat it now at a lower risk/volume category with higher cure rate chances and a young age to make a good functional recovery, and (2) take advantage of the next 3‐5 years on active surveillance free of treatment‐related side effects. There is no right or wrong approach here. The dilemma is sort of a “pay it now or pay it later” situation, but I have also used the phrase “beauty is in the eye of the beholder,” to illustrate the point that some active surveillance vs treatment decisions are subjective, and patients may look at the value of each strategy differently.

For another bit of a rabbit trail diversion, the phrase “beauty is in the eye of the beholder” is credited to an author Margaret Wolfe Hunderford from her book in 1878. It is also an album and track title from 1988 from one of my favourite jazz‐fusion artists Chick Corea. I was the bass player for the Davidson College Jazz Band from 1986–1990 and enjoyed playing works from Corea and his various band and solo projects. I have always wondered how jazz artists and other pure instrumentalists come up with album and track titles when there are no song lyrics, repeating choruses, or stories being told. Perhaps the concepts are only broad in nature and Corea works the phrasing with a separate track called “Beauty” (my favourite—a good tune for the OR), and “Eye of the Beholder,” “Passage,” “Forgotten Past,” “Amnesia,” and others. I recommend you check it out—a true fusion‐jazz concept with powerful rhythms, creative structures, solid melodies, and a lot of instrumental improvisation. I outlined several parts of this editorial in January of this year only to learn of Corea's passing on February 9th, 2021 at the age of 79. We lost an amazingly talented and pioneering musician and happy to pay him tribute with this article. His career goes back to the 1960s and included 23 Grammy Awards.

Moving on to our March 2021 issue with nine articles of open access content, and authorship groups from the United States, Canada, Australia, Japan, and the United Kingdom. Please enjoy Figures 1, 2, 3, 4, 5, ^1^ as photographic tributes to the beautiful scenes from our authors’ countries of origin.

- We have a **research communication** from Eleswarapu et al from UCLA in Los Angeles,^3^ USA. This work is an interesting focus and set of methods for researching online message boards as a source for patient information for prostate cancer. Have a look at their use of “meaning extraction method” and see if it may give you ideas for your own future studies.
- **To the Journals…**The review by Xi et al^4^ is a systematic methods effort that focused on the question of whether or not hydrophilic‐coated versus standard catheters are cost‐effective in spinal cord injury patients. Of eight studies that passed eligibility, five supported hydrophilic‐coating, and the authors dive into the details of the conflicting results with an overall favourable rating of the cost‐effective and quality of life merits.
- **To the Clinic…**We have three articles in this section. Merrick et al^5^ have the themed article mentioned above on active surveillance. This is an interesting cohort with all patients staged with a transperineal mapping biopsy—hoping to exclude under‐graded patients. The interesting stat line is the therapeutic intervention rates at 10 years of 0% for ages under 60, 1% for ages 60–69, and 11.4% for ages over 69. Their mean time to intervention was just under 5 years—so overall a good series to use and discuss with patients how they want to solve their “eye of the beholder” choices between early intervention and surveillance with possible delayed intervention. Moving on, we have two Covid‐19 era‐related papers to help your clinical decision making. Farag et al^6^ look at ureteric calculi management in an Australian setting, and Stroman et al^7^ looked at an institution‐wide approach to elective surgeries carried out in a “cold” unit with testing and pre‐admission self‐isolation, while acute admissions stayed in a separate “hot” site.
- **To the Drawing Board…** Bhatt et al^8^ present a survey study on whether or not stenting is needed after uncomplicated ureteroscopy. However, it is really a prelude to feasibility for a randomized trial—basically a feasibility survey study. They demonstrated a wide variation in practice and are moving forward with their trial. This is a nice feature for us to publish and we were pleased to see a lot of social media activity on this one from the @BURSTUrology group. Miyoshi et al^9^ from Japan present their work on a prognostic model for patients with bone‐metastatic hormone‐naïve prostate cancer. This is an extension of the CHAARTED trial risk classification and identifies four risk groupings to further stratify survival and need for upfront combination systemic therapies.
- **To the Future…** Finally, we finish with a multi‐institutional randomised trial on a new technology in prostate ultrasound called micro‐ultrasound which has a high frequency 29 MHz probe. The paper by Pavlovich et al^10^ is an early experience and includes rich information on how the system can be used and interpreted for regions of interest, similar to MRI. In this study, the trial showed no benefit over conventional ultrasound, but there were upward trends with increasing experience. From what I have heard at various meetings, this is an interesting technology to keep an eye on that may be of use for our prostate cancer screening and active surveillance populations. See our YouTube channel for a video presentation on this work.

**FIGURE 1:**
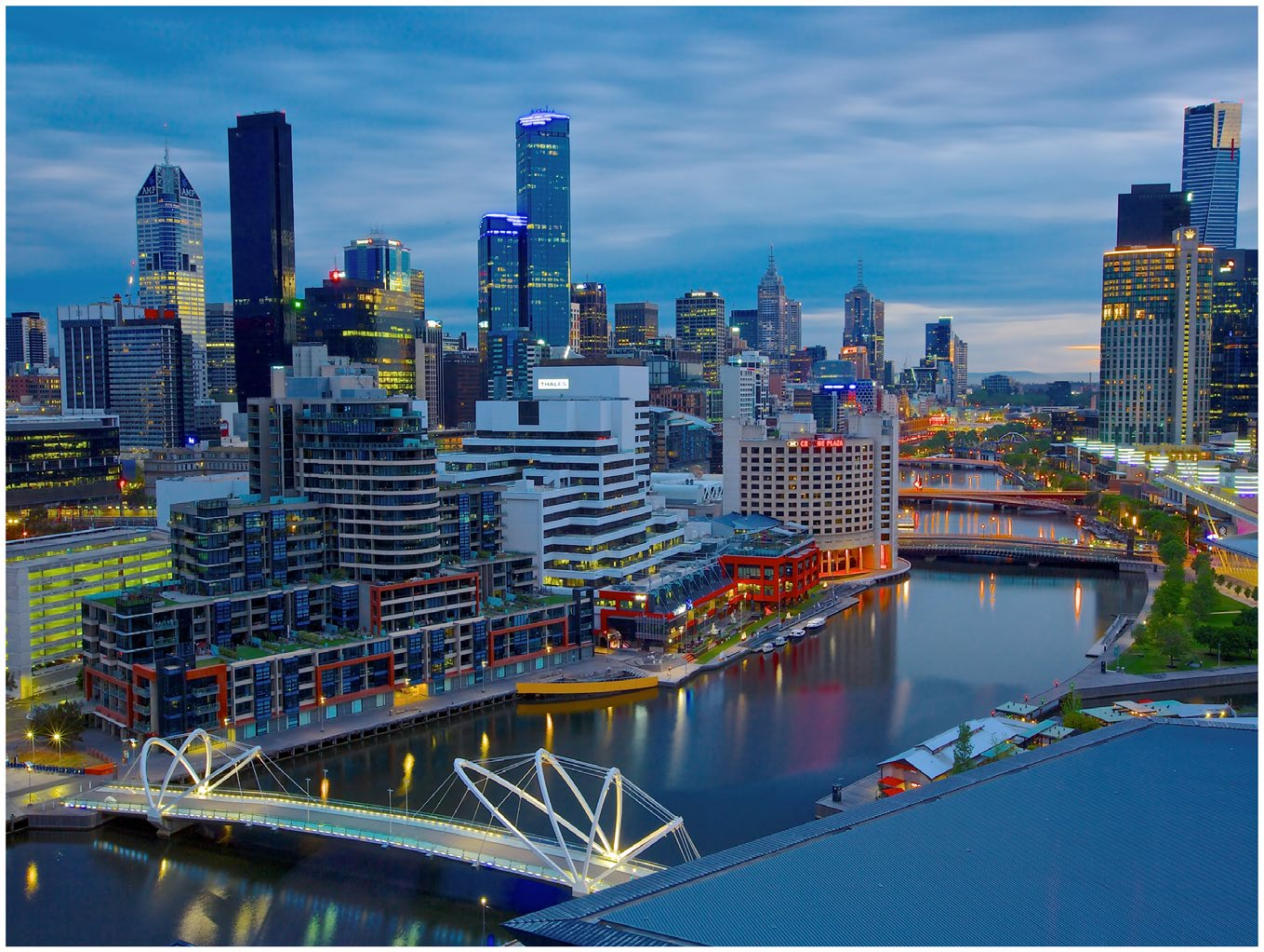
From Australia: a beautiful morning in Melbourne along the Yarra River

**FIGURE 2:**
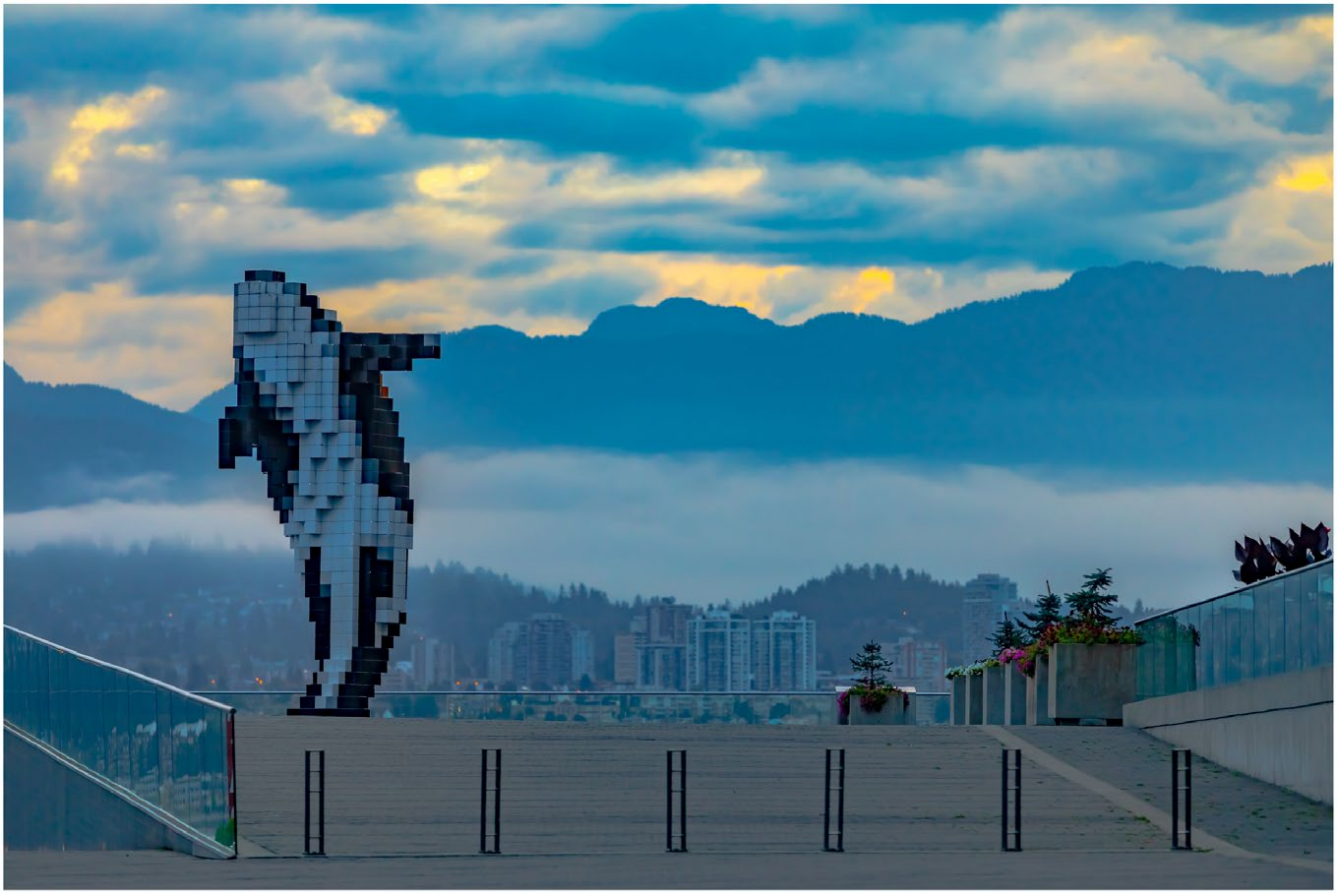
From Canada: beauty in the Vancouver combination of urban living and the rugged Pacific Northwestern mountains

**FIGURE 3:**
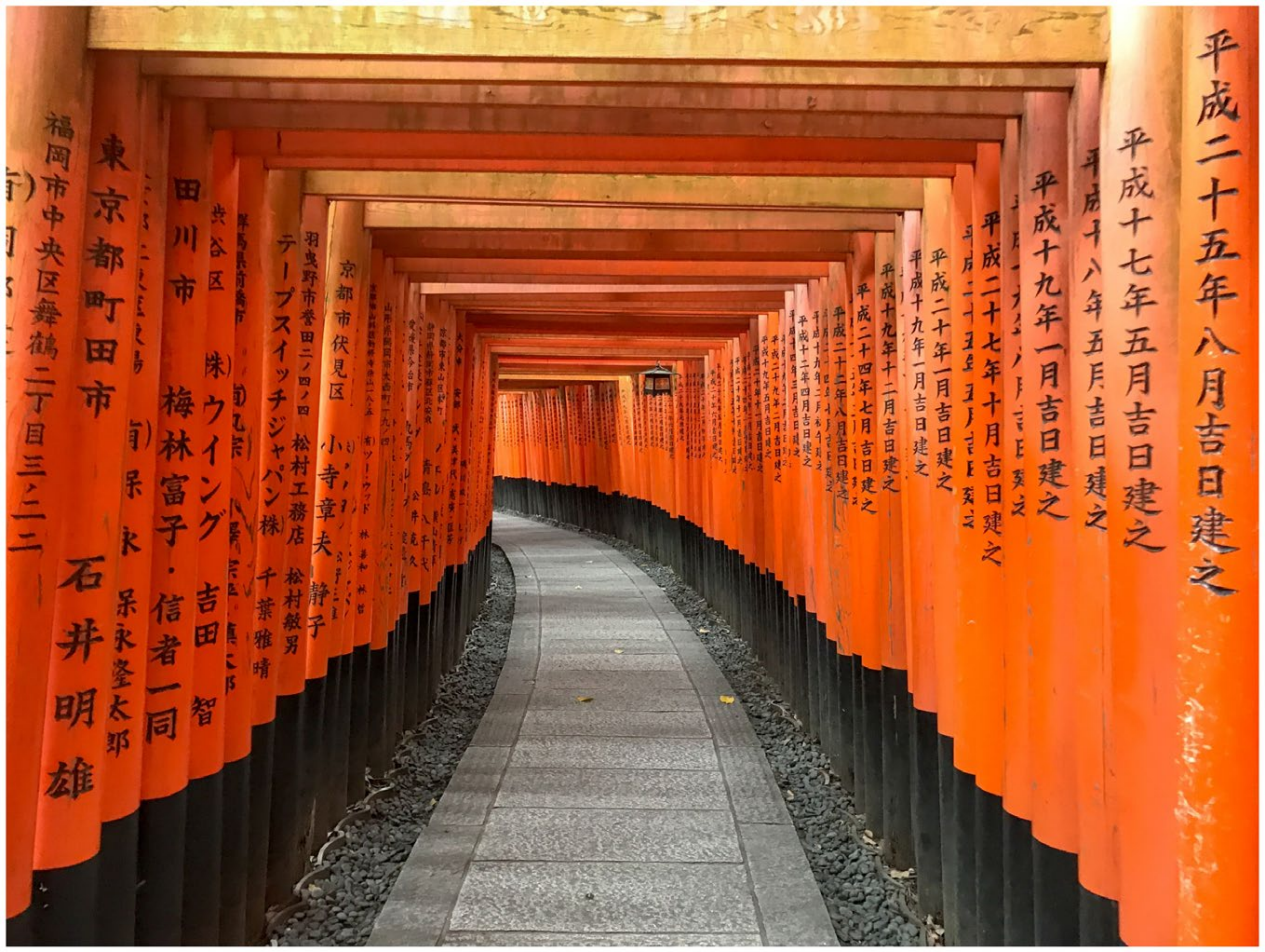
From Japan: the most “instagramable” travel photo from Fushimi Inari Taisha Shrine in Kyoto

**FIGURE 4:**
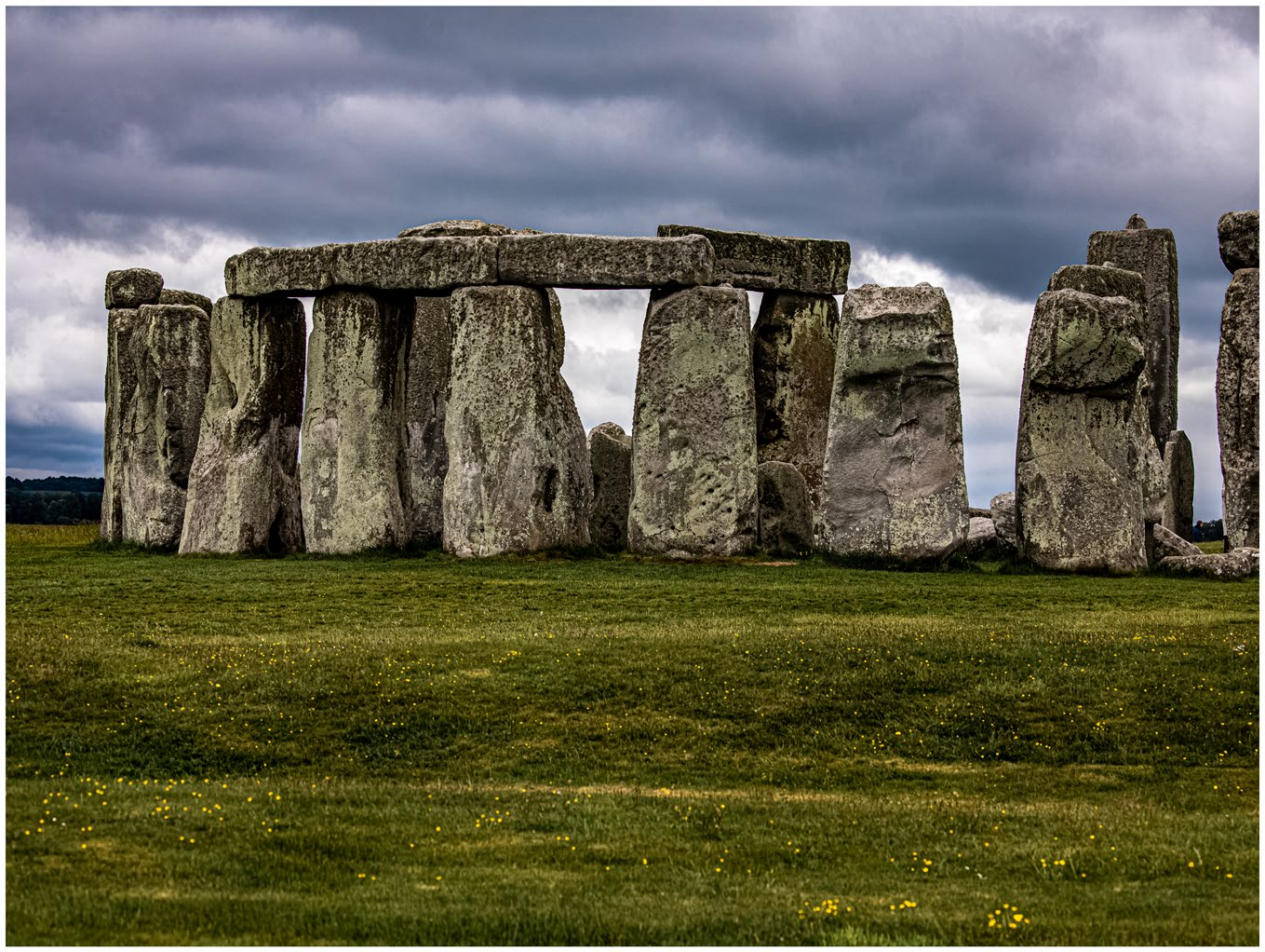
From the United Kingdom: beauty and mystery surrounding Stonehenge

**FIGURE 5:**
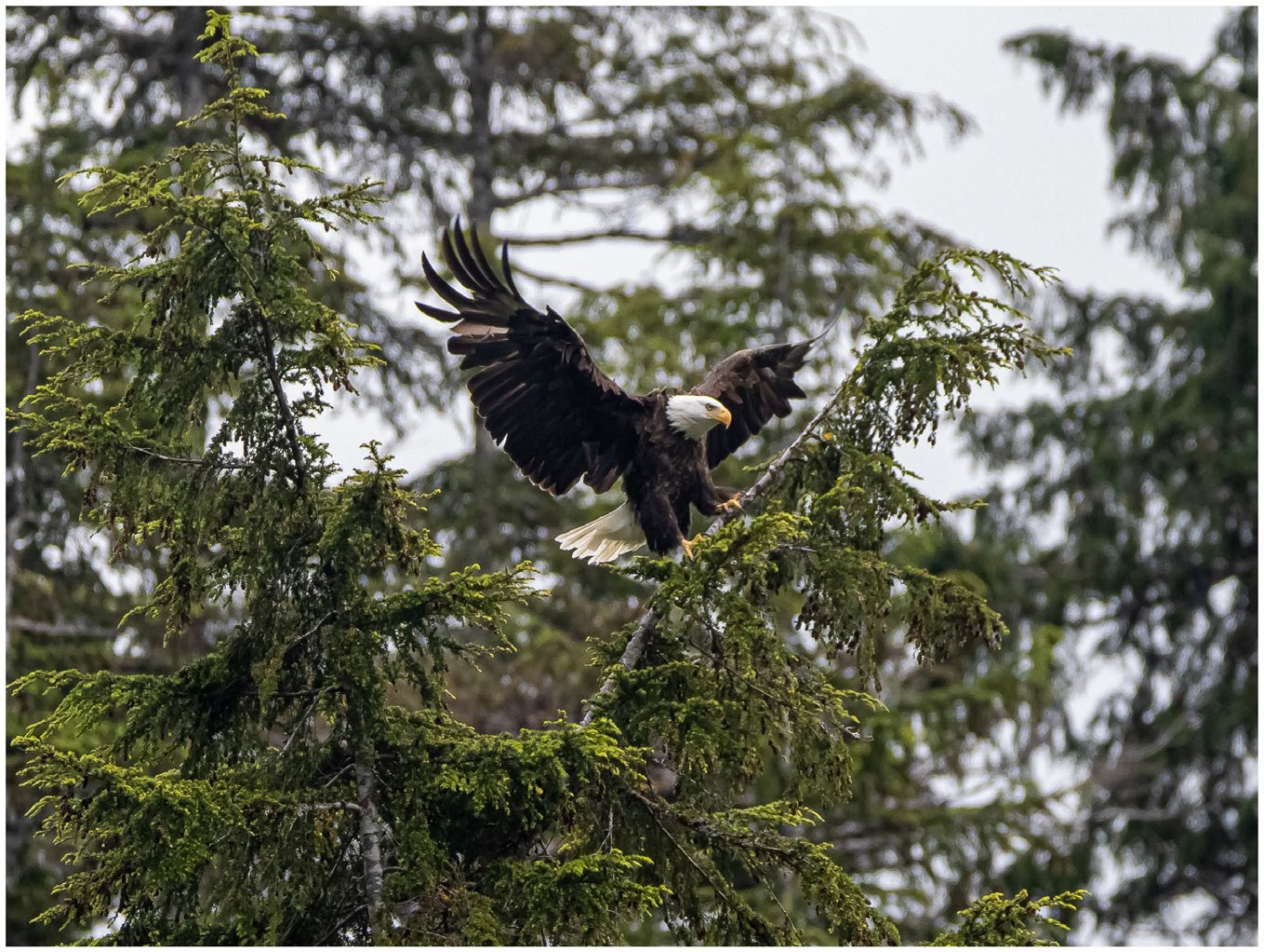
The bald eagles fly and fish in the protected areas of the Alaskan Inside Passage

## ENDNOTE

^1^
Photography by John W. Davis, MD, FACS.
